# Development of a novel secondary phenotypic screen to identify hits within the mycobacterial protein synthesis pipeline

**DOI:** 10.1096/fba.2020-00022

**Published:** 2020-08-20

**Authors:** Christopher Burke, Monika Jankute, Patrick Moynihan, Ruben Gonzalez del Rio, Xiaojun Li, Jorge Esquivias, Joël Lelièvre, Jonathan A. G. Cox, James Sacchettini, Gurdyal S. Besra

**Affiliations:** ^1^ Institute of Microbiology and Infection School of Biosciences University of Birmingham Birmingham UK; ^2^ Diseases of the Developing World GlaxoSmithKline Tres Cantos Madrid Spain; ^3^ Department of Biochemistry and Biophysics Texas A&M University College Station Texas United States; ^4^ School of Life & Health Sciences Aston University Birmingham UK

**Keywords:** mCherry, mycobacteria, ribosome, RNA polymerase, transcription, translation

## Abstract

**Background:**

Whole‐cell phenotypic screening is the driving force behind modern anti‐tubercular drug discovery efforts. Focus has shifted from screening for bactericidal scaffolds to screens incorporating target deconvolution. Target‐based screening aims to direct drug discovery toward known effective targets and avoid investing resources into unproductive lines of enquiry. The protein synthesis pipeline, including RNA polymerase and the ribosome, is a clinically proven target in *Mycobacterium tuberculosis*. Screening for new hits of this effective target pathway is an invaluable tool in the drug discovery arsenal.

**Methods:**

Using *M*.* tuberculosis* H37Rv augmented with anhydrotetracycline‐inducible expression of mCherry, a phenotypic screen was developed for the identification of protein synthesis inhibitors in a medium throughput screening format.

**Results:**

The assay was validated using known inhibitors of protein synthesis to show a dose‐dependent reduction in mCherry fluorescence. This was expanded to a proprietary screen of hypothetical protein synthesis hits and modified to include quantitative viability measurement of cells using resazurin.

**Conclusion:**

Following the success of the proprietary screen, a larger scale screen of the GlaxoSmithKline anti‐tubercular library containing 2799 compounds was conducted. Combined single shot and dose‐response screening yielded 18 hits, 0.64% of all screened compounds.

## 1 INTRODUCTION

*Mycobacterium tuberculosis*, the causative agent of tuberculosis (TB), remains one of the most successful bacterial pathogens. Despite extensive international co‐operation TB remains extremely difficult to treat and has a high mortality rate, with an estimated 10.4 million new cases and over 1.7 million deaths in 2016 alone.^1^ The long duration and unpleasant side effects of TB treatment regimens results in poor patient compliance that together with global overuse of antibiotics have contributed to the rise of multidrug resistant (MDR) and extensively drug resistant (XDR) strains of *M*.* tuberculosis*. In 2016, there were approximately 600 000 rifampicin and MDR TB new cases, leading to 240 000 deaths worldwide.^2^ In order to address the need for new antimicrobials and help combat the rising issue of antibiotic resistance, drugs with novel modes of action against clinically proven targets need to be identified.

The protein synthesis pipeline, containing RNA polymerase (RNAP), approximately 22 tRNA synthases,^3^ and the ribosome represents one of the most crucial aspects of biological life. Both RNAP and the ribosome have been successfully inhibited in *M*.* tuberculosis* by rifampicin (RIF)^4^, ^5^, ^6^, ^7^, ^8^ and streptomycin,^9^, ^10^, ^11^, ^12^, ^13^, ^14^ respectively, drugs that see extensive use in the clinic for treating TB.^15^ New scaffolds that target these complexes at different sites are an attractive proposition for further drug development and clinical use. Recently, Lin et al demonstrated compounds that inhibit RNAP at an alternate site to RIF, can be co‐administered, and have a cumulative bactericidal effect resulting in suppression of antibiotic resistance generation in *M*. *tuberculosis*.^16^ Several ribosomal inhibitors may also have synergistic action when used together. Linezolid, a ribosomal inhibitor with unfavorable side effects, has increased bioavailability when co‐administered with clarithromycin, allowing a reduction in dose delivered to patients with MDR‐TB.^17^ In addition, spectinomycin displays a reduced minimum inhibitory concentration (MIC) in combination with other mycobacterial ribosomal inhibitors from the macrolide, tetracycline, and lincosamide classes.^18^, ^19^ While, there are no commercially approved tRNA synthase inhibitors for the treatment of TB, several inhibitors have been outlined in the literature.^20^, ^21^, ^22^, ^23^ Specifically, the 3‐aminomethyl compounds containing boron that successfully inhibit mycobacterial leucyl‐tRNA synthetase,^21^, ^22^ as well as, various inhibitors of aspartyl‐tRNA synthetase.^20^, ^23^

**Figure 1:**
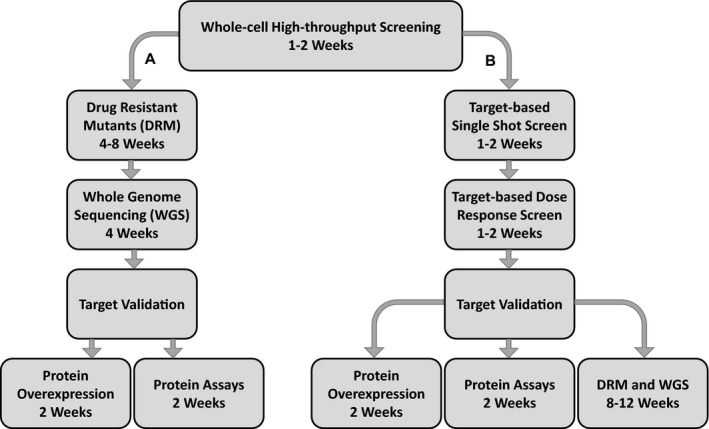
Standard drug target validation pathway (A) and target‐based screening pathway (B). The standard method of target validation uses DRM generation and WGS to identify targets, which can be time‐consuming and result in identification of non‐favorable drug targets. Target‐based screening cuts the time investment to target identification and shifts DRM generation and WGS to a position where they can be conducted concurrently with other target validation steps

Drug discovery and target identification is a laborious process that is bottlenecked at several key points. The current reliance on generation of spontaneous drug‐resistant mutants (DRMs) and whole genome sequencing (WGS) of single nucleotide polymorphisms (SNPs) and deletions is effective,^20^, ^24^, ^25^, ^26^ but has a high failure rate. In addition, not all compounds are suitable for the generation of DRMs as the target may be highly conserved and not prone to mutation(s). Additionally, it is not uncommon for SNPs to appear “off target” from the gene of interest.^27^ By incorporating target‐based methods into screening, compounds can be sifted for a specific mode of action prior to DRM generation and WGS. More specific biochemical assays can then be conducted in parallel with DRM generation and WGS. Figure 1 compares a more traditional DRM‐driven target validation against the target‐based screening.

Here, a method was developed to identify potential protein synthesis hits from compound libraries using whole‐cell phenotypic screening as a tool. This screen in addition to other, recently developed,^28^, ^29^ whole‐cell phenotypic screening methods is focused on reducing the time required for mode of action studies into known effective targets.

## 2 MATERIALS AND METHODS

### 2.1 Bacterial strains and growth conditions

*M*.* tuberculosis* H37Rv strain^27^ was electroporated with a kanamycin‐resistant mycobacterial plasmid pTIC6a‐*mCherry* ^30^ and transformants selected on Middlebrook 7H10 media containing 10% (v/v) OADC and 25 μg/mL of kanamycin at 37°C with 5% CO_2_. Single colonies were inoculated into 10 mL of Middlebrook 7H9 containing 10% OADC (v/v), 0.2% (v/v) glycerol, 0.05% (v/v) Tween‐80 or Tyloxapol and 25 μg/mL of kanamycin, and statically cultured at 37°C with 5% CO_2_ for ~7 days. The expression of *mCherry* gene was induced with the addition of 100 ng/mL of anhydrotetracycline. Growth rate was monitored by measuring optical density (OD) at 600 nm and cells were passaged to maintain a mid‐log culture at OD_600_ of 0.4–0.6. To prevent mutagenesis, no more than four passages were conducted before cultures were reinitiated from a parent glycerol stock. For all assays conducted no greater than 2% DMSO was ever used. MICs for known protein synthesis inhibitors (apramycin, streptomycin, chloramphenicol, and hygromycin) and controls (isoniazid and ethambutol) were determined in flat‐clear bottom 96‐well plates (Greiner, 655090). Isoniazid, ethambutol, chloramphenicol, and hygromycin were dissolved in DMSO and apramycin and streptomycin in 0.3% (v/v) Tween‐20, in both cases 2% of the total assay volume. *M*.* tuberculosis* H37Rv was grown as above without kanamycin to OD 0.4 and diluted to 5 × 10^5^ cells/mL. Diluted cell culture (90 µL), test compound (10 µL) was inoculated into each well up to make a final 100 μL solution. Plates were incubated for 7 days at 37°C with 5% CO_2_. After incubation, 30 μL of 0.02% resazurin and 12 μL of 20% Tween‐80 were added to each well and incubated for a further 24 hours and then visually observed to determine the compound MICs. HepG2 cytotoxicity was performed using HepG2 cells cultured using Eagle's minimum essential medium supplemented with 10% heat‐inactivated fetal bovine serum, 1% non‐essential amino acids, and 1% penicillin/streptomycin as described previously.^26^

### 2.2 Construction of pTIC6a‐*mCherry* vector

A codon‐optimized variant of *mCherry* was purchased from Genscript and the full‐length mCherry gene was amplified using the following primer pair (restriction sites underlined): *mCherry*Fwd (5’‐GAG GAA GCT TAT GGT GAG CA‐3’) and *mCherry*Rev (5′‐TGT ACA AGT GAG AAT TCA TA‐3′). The PCR product was digested with *Hin*dIII and *Eco*RI and subsequently ligated into pTIC6a digested with identical enzymes. DNA sequencing and construct verification was carried out at the Eurofins DNA Ltd.

### 2.3 Validation of the mCherry reporter screen

Known protein synthesis inhibitors (apramycin, streptomycin, chloramphenicol, and hygromycin) and controls (isoniazid and ethambutol) were dispensed into 384‐well black wall flat‐clear bottom plates (Greiner, 78109) in a dose‐response fashion using HP Digital Dispenser. Isoniazid, ethambutol, chloramphenicol, and hygromycin were dissolved in DMSO and apramycin and streptomycin in 0.3% (v/v) Tween‐20, in both cases 2% of the total assay volume. Mid‐log phase *M*.* tuberculosis* H37Rv::pTIC6a‐*mCherry* cells were pelleted by centrifugation at 4000 rpm for 15 minutes and then, washed twice with fresh Middlebrook 7H9 media to remove traces of kanamycin and adjusted to an OD_600_ of 0.4 in Middlebrook 7H9 media without kanamycin. The culture was then split into two: one was left un‐induced (‐anhydrotetracycline) and the second was induced with 100 ng/mL anhydrotetracycline (+anhydrotetracycline). Plate wells received cells at an OD_600_ 0.4 and in a final volume of 50 μL and were incubated to 37°C with 5% CO_2_ and left for 48 hours. Readings of mCherry fluorescence were taken using a PHERAstar FS from BMG LabTech plate reader at excitation 580 nm/emission 620 nm. After reading mCherry fluorescence, 15 μL of 0.02% resazurin and 6 μL of 20% Tween‐80 were added to each well and the plates were incubated for a further 24 hours. Plates were visually inspected for cellular viability based upon resazurin color change. Experiments were performed in triplicate.

### 2.4 Preliminary mCherry reporter screen using *M*.* tuberculosis* ribosome hits

The 38 compounds identified as *M*.* tuberculosis* ribosome inhibitors in a separate study (unpublished data, Professor James Sacchettini from Texas A&M University) were solubilized in 100% DMSO and automatically dispensed into 384‐well black wall flat‐clear bottom plates (Greiner, 78109) in a dose‐response fashion using an Echo Acoustic dispenser from Labcyte, to afford a final 0.25 µL solution. *M*.* tuberculosis* H37Rv::pTIC6a‐*mCherry* cells were treated as described above and both +anhydrotetracycline and –anhydrotetracycline culture stocks of OD_600_ of 0.4 were dispensed into appropriate wells to a final volume of 50 µL. Plates were incubated and fluorescence of mCherry was measured as described above. Resazurin was added to plates as above and fluorescence measurements were taken using plate reader PHERAstar FS from BMG LabTech at excitation 530/emission 590 nm.

### 2.5 Combined single shot screen and dose‐response screens of GSK TB box

*M*.* tuberculosis* H37Rv::*mCherry*‐pTIC6a cells were prepared as described above. The cell suspension was then supplemented with 100 ng/mL of anhydrotetracycline. The screening and dose‐response experiments were conducted in 384‐well black wall flat‐clear bottom plates (Greiner, 78109). The single shot screen assessed two different concentrations of drug, 10 μmol/L and 1 μmol/L, at a final volume of 50 μL in duplicate. The subsequent dose‐response confirmation screen had a range of concentrations spanning 50 μmol/L to 0.846 nmol/L at a final volume of 50 μL using four replicate plates. The compounds in both screens were dispensed into plates using an Echo Acoustic Dispenser by GSK. Positive control wells included 0.5% DMSO only, whereas negative control wells had 6.24 μg/mL of hygromycin, four times the MIC. Plates contained 50 μL of cells at an OD_600_ of 0.4 containing anhydrotetracycline added to each well. Plates were read on a PHERAstar FS plate reader at excitation 580 nm/emission 620 nm for mCherry fluorescence after 60 hours of incubation at 37°C with 5% CO_2_. Cell viability was assessed using resazurin as per above using a PHERAstar FS plate reader at excitation 530 nm/emission 590 nm.

### 2.6 Z’ statistics and percentage survival calculation

Each plate had *Z′* calculation conducted upon it using the equation –

$$Z^{\prime }=1‐\frac{3\left(\sigma_{p}+\sigma_{n}\right)}{\left(\mu_{p}‐\mu_{n}\right)}$$

where *σ*_p_ and *σ*_n_ equal the standard deviation of the positive and negative controls, respectively, and *μ*_p_ and *μ*_n_ equal the mean of the positive and negative controls. Plates with a Z prime of less than 0.5 were considered to have poorly separated controls and were excluded from the experiments. Results were then converted to percentage mCherry expression as per the equation –

$$\%\text{mCherryExpression}=\left(\frac{x‐\hat{n}}{\hat{p}‐\hat{n}}\right)\times 100$$

where x equals the experimental value, $\hat{p}$ equals the mean of the positive control,s and $\hat{n}$ equals the mean of the negative controls.

### 2.7 Broken beacon RNA polymerase assay

The broken beacon RNA polymerase assay^31^ uses a 5′ TAMRA labeled DNA oligo that hybridizes to a shorter 3’ BHQ2 labeled DNA oligo. When hybridized the BHQ2 effectively quenches the TAMRA fluorophore. When the hybridized probes encounter complimentary RNA produced by RNA polymerase, the shorter BHQ2 probe dehybridizes and the longer RNA product and TAMRA probe hybridize. This removes the BHQ2 from the proximity of the TAMRA and the resulting increase in fluorescence can be detected. BHQ2 and TAMRA oligos were hybridized in a 2:1 ratio, 8 μmol/L:4 μmol/L in a 500 μL aliquot. BHQ2 oligo (40 μL of 100 μmol/L), TAMRA oligo (20 μL of 100 μmol/L), 100 μL 5× SSPE buffer, 25 μL 20× Denhardt's reagent, and 315 μL of dH2O were added into a 1.5‐mL microcentrifuge tube. The mixture was heated at 95°C which reduced by 10 °C every 5 minutes. Reactions of 20 μL were set up with *E. coli* RNA Polymerase, Holoenzyme (NEB, M0551S). RNA polymerase buffer (5×, 4 μL), 1 μL *E*. *coli* RNA polymerase, 125 μmol/L ATP, 125 μmol/L CTP, 125 μmol/L GTP, 125 μmol/L UTP, 5 μL of hybridized TAMRA/BHQ2 oligos, 40 nM of *mmsA* DNA template were made up to 19 μL with RNase free dH_2_O. Hits in 20% DMSO (2 mmol/L) were added for 100 μmol/L compound in 1% DMSO. The reactions were read on a BMG plate reader every 30 seconds for 1000 cycles. The sequence of the TAMRA and BHQ2 probes are shown in Table 1. The *mmsA* template DNA was 440 base pairs long and included 179 bases pairs of DNA upstream of the *mmsA* start site to include the promotor region. The primers for the PCR of the *mmsA* template are shown in Table 2.

**Table 1:** DNA sequence of broken beacon probes and location of attached modifications. TAMRA is excited at a wavelength of 557 nm and emits at a wavelength of 583 nm. BHQ2 maximally quenches at 579 nm.

DNA Sequence 5′ – 3′	Modifications
ttcacatttcatcgacggacaacg	5′ – TAMRA
cgatgaaatgtgaa	3′ – BHQ2

**Table 2:** Primers for *mmsA* template DNA.

Direction	DNA Sequence 5′ – 3′
Forward	catgcatgcatatgctagccatgatggagcgcag
Reverse	catgcatgaagcttcagctcggccaactcgtcga

## 3 RESULTS

### 3.1 Development of a phenotypic screening assay to identify protein synthesis hits

**Figure 2:**
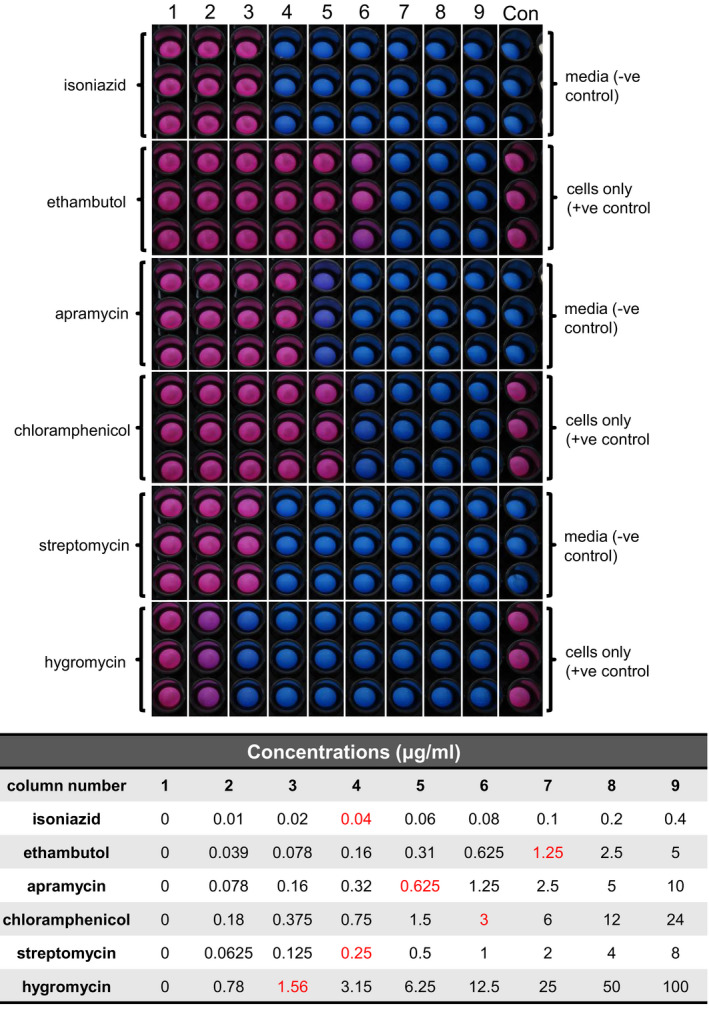
MIC determination of known protein synthesis inhibitors and control inhibitors. Visual confirmation of drugs MICs using resazurin. Pink colored wells indicate cell survival. Blue wells indicate cell death. Red values in the table show the MIC for each compound

**Figure 3:**
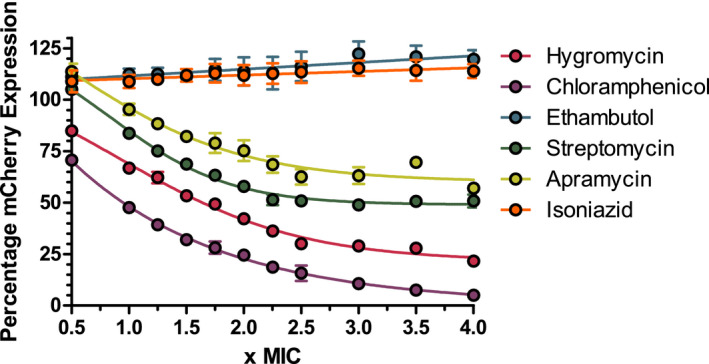
The effect of known inhibitors on the percentage expression of mCherry. Isoniazid and ethambutol negative controls show no mCherry expression decrease. Known protein synthesis inhibitors hygromycin, chloramphenicol, streptomycin, and apramycin show a dose‐dependent decrease in mCherry expression

Target identification is a crucial step in TB drug discovery, which is commonly conducted using WGS of spontaneous DRMs. The caveat of this process is that discovered targets may not be suitable or may not be the true target at all. To avoid these pitfalls, whole‐cell phenotypic screening to search for molecules inhibiting specific targets from a compound library has become common place. A phenotypic screen utilizing mCherry fluorescence to identify hits of the protein synthesis pipeline was developed as there are several key enzymes that have already been clinically validated as excellent targets for drug treatment within this pathway. The method uses anhydrotetracycline‐inducible expression of the fluorescent protein mCherry as a read‐out for whole‐cell protein expression. To validate this approach, several known inhibitors of the ribosome were used to assess drug‐mediated protein expression inhibition and therefore fluorescence reduction. The MICs of these drugs was determined and is shown in Figure 2 and Table 3. Crucial to this approach is the ability to monitor cell survival to ensure that the fluorescence is not reduced simply from cell death. The data shown in Figure 3 show that the known protein synthesis inhibitors—chloramphenicol, streptomycin, apramycin, and hygromycin—display a dose‐dependent reduction in protein synthesis expression, which can be measured using the mCherry fluorescent protein. In addition, the cellwall synthesis inhibitors—isoniazid and ethambutol—showed no decrease in mCherry fluorescence. Fluorescence was converted to percentage mCherry expression. The positive control used was 2% DMSO with anhydrotetracycline to induce mCherry expression and the negative control was without anhydrotetracycline to account for background leaky protein expression. The short incubation period resulted in a detectable reduction in protein synthesis without causing cellular death. To ensure the reduction in protein synthesis was not due to reduced cell viability, resazurin was used to visually assess cell survival after measuring mCherry fluorescence.

**Table 3:** MIC values of known inhibitors used to validate mCherry screen. These concentrations correspond to the x MIC values for Figure 3.

MIC (μg/mL)	×0.5	×1	×1.25	×1.5	×1.75	×2	×2.25	×2.5	×3	×3.5	×4
Isoniazid	0.02	0.04	0.05	0.06	0.07	0.08	0.09	0.10	0.12	0.14	0.16
Ethambutol	0.63	1.25	1.56	1.88	2.19	2.5	2.81	3.13	3.75	4.38	5.00
Apramycin	0.31	0.63	0.78	0.94	1.09	1.25	1.41	1.56	1.88	2.19	2.50
Chloramphenicol	1.50	3.00	3.75	4.50	5.25	6.00	6.75	7.50	9.00	10.50	12.00
Streptomycin	0.13	0.25	0.31	0.38	0.44	0.50	0.56	0.63	0.75	0.88	1.00
Hygromycin	0.78	1.56	1.95	2.34	2.73	3.12	3.51	3.90	4.68	5.46	6.24

### 3.2 Proprietary screen with *M*.* tuberculosis* ribosome inhibitors

**Figure 4:**
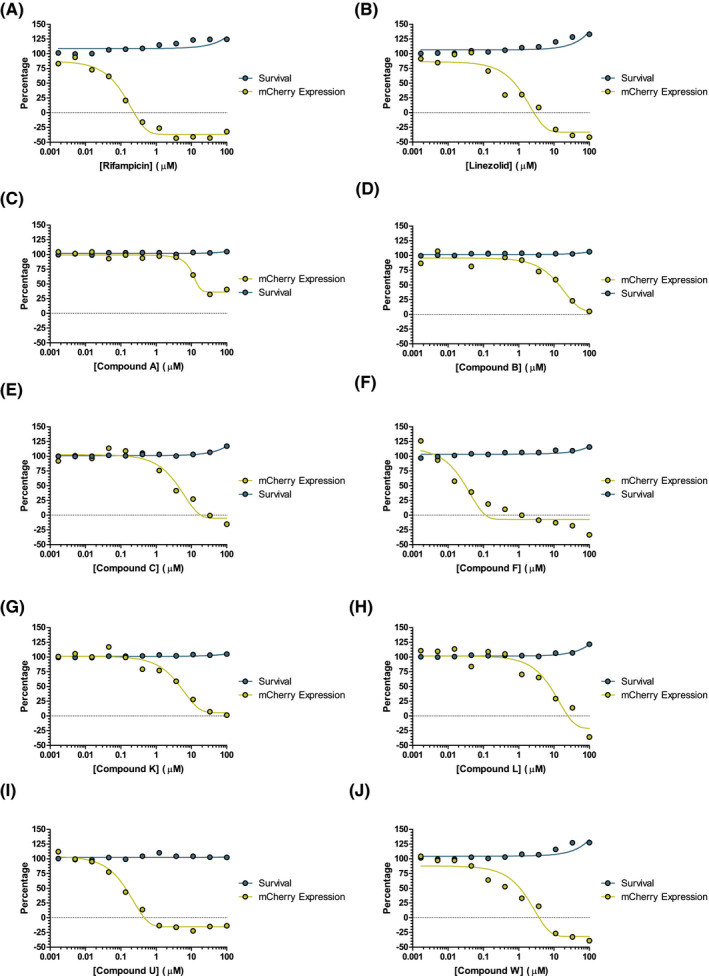
Dose response of hit compounds found in the preliminary screen. Percentage mCherry expression displayed in yellow. Percentage cell survival displayed in blue

Initial screening focused on a select group of 38 hits that targeted the ribosome (Table 4) provided by Professor James Sacchettini, established using a cell‐free method (unpublished data). Of the 38 compounds, 23 showed a dose‐dependent reduction in mCherry expression consistent with protein synthesis inhibition. Importantly, resazurin fluorescence showed that the decrease in mCherry expression across the plates was due to protein synthesis inhibition as indicated by decrease in mCherry expression and not due to a reduction in cellular survival. The data in Figure 4 show 10 of the compounds that displayed a dose response. These also include RIF and linezolid, which were used as controls. The compounds that failed to show a dose‐dependent reduction in mCherry expression were potentially unable to pass through the mycobacterial outer membrane.

**Table 4:** Molecular structures of proprietary screen hits.

Compound	Molecular Structure
A	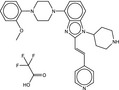
B	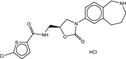
C	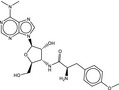
F	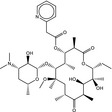
K	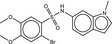
L	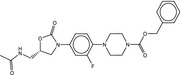
U	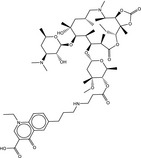
W	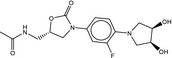

### 3.3 Single shot high throughput screen results

**Figure 5:**
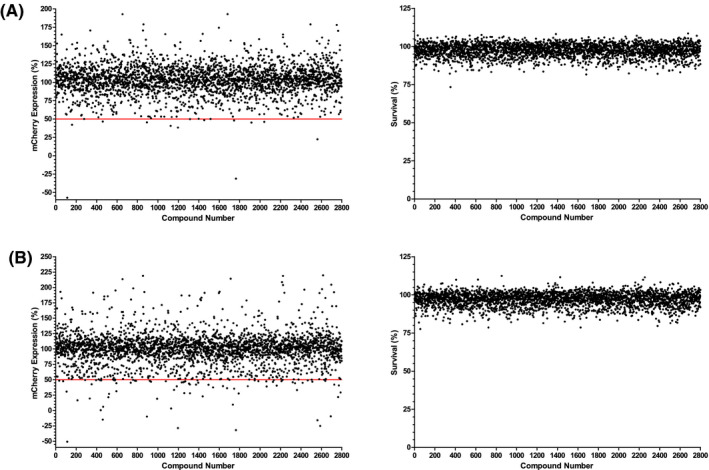
Percentage expression results and cell survival for all 2799 compounds screened. The red line denotes the cutoff threshold for positive results displaying lower than 50% mCherry expression. Panel A shows results for the 1 μmol/L screen, panel B shows the 10 μmol/L screen. The left panels of each figure show the percentage mCherry expression and the right panels show the percentage survival as determined by resazurin

The single shot screen, shown in Figure 5, was used as a tool for separating potential hits from a larger chemical library. The compounds screened were the TB box‐set library produced by GlaxoSmithKline (GSK) of anti‐tubercular drugs.^31^, ^32^ Single well experiments were used at two compound concentrations, 10 μmol/L and 1 μmol/L, and potential hits were designated by a percentage mCherry expression of less than 50% at either 10 μmol/L or at both 10 μmol/L and 1 μmol/L. Fluorescence values were converted to percentage mCherry expression, as *per* the validation, by subtracting the hygromycin negative control and normalizing against the DMSO‐positive control. It should be noted that in Figure 5A,B at higher concentrations, it may well be possible that some hits may produce a detergent‐like effect and stimulate growth. After treatment with resazurin, the cells in the individual experiments were checked for viability to assure a decrease in mCherry fluorescence was due to protein synthesis inhibition and not due to cell death. Of the 2799 compounds screened, 91 compounds, 3.25%, were designated as hits.

### 3.4 Dose‐response results

**Figure 6:**
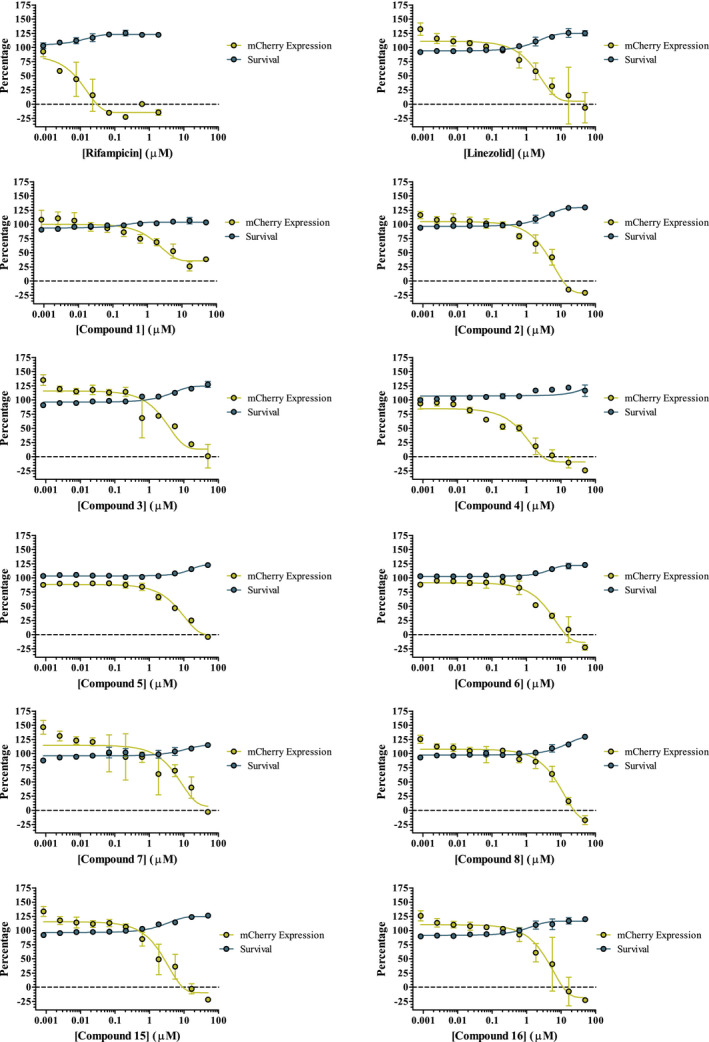
Dose response of hit compounds found in the large‐scale screen. Percentage mCherry expression displayed in yellow. Percentage cell survival displayed in blue. Compound 2 was an additional blind linezolid control

Compounds identified by the single shot screen were subjected to a more rigorous dose‐response testing to filter out any false positives. Using secondary dose‐response screening allows the selection criteria in the single shot screen to be more relaxed. This can help account for genuine hits that may normally be missed due to having a standardized compound concentration, that is, where the compound concentration is too low to show the desired phenotype. The range of concentrations spanned 50 μmol/L to 0.846 nmol/L, this started higher and ended lower than the concentrations used in the single shot screen. The plates were first assessed by *Z′*, any plate with a *Z′* lower than 0.5 was excluded from the results. This resulted in one group of plates having results in triplicate instead of quadruplicate. The average *Z′* across the whole experiment, including the excluded plate, was 0.626. The fluorescence values were normalized as described above and of the ninety one initial hits progressed, 18 displayed a desired dose‐dependent response, 10 of which are shown in Figure 6 and Table 5, in addition to RIF and linezolid included in the screen as controls. This represented 0.64% of all compounds screened.

**Table 5:** Molecular structures of TB box screen hits.

Compound	Molecular Structure	HepG2 Tox50 (μmol/L)
1	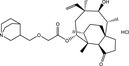	4
2 (Linezolid)	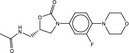	4
3	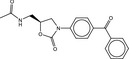	4
4	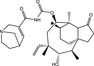	4
5	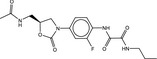	4
6	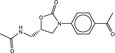	4.1
7	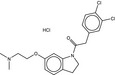	4
8	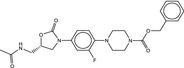	4
15	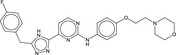	4.4
16	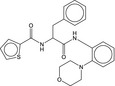	4

### 3.5 Broken beacon RNA polymerase assay results

**Figure 7:**
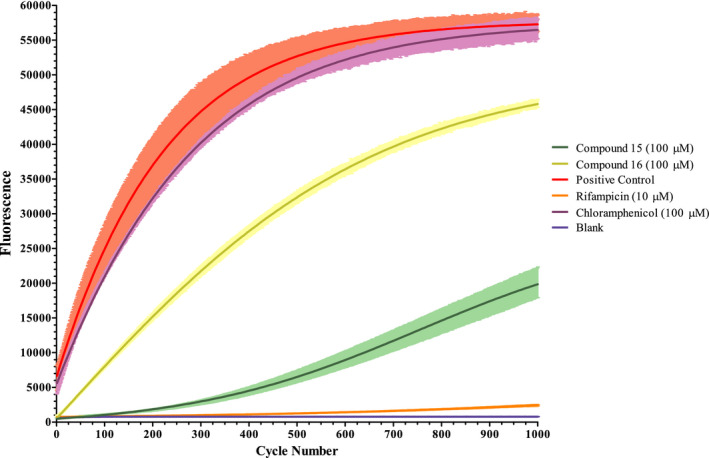
Broken beacon RNA polymerase assay. The plotted lines denote the average fluorescence values from three test wells from 1000 read cycles of 30 seconds. Error bars are shown in lighter colors. The blank shows the base fluorescence from probe dehybridization

To validate this screening methodology, 10 of the 18 dose‐response hit compounds were examined for activity in an RNA polymerase assay using commercial *E*.* coli* RNA polymerase. The hits were screened along with positive and negative control inhibitors rifampicin (RNA polymerase) and chloramphenicol (protein synthesis inhibitor), respectively. The results of two of the hits are shown in Figure 7. The positive control and blank sample show good separation, with rifampicin knocking activity down to near blank levels and chloramphenicol showing negligible effect on overall activity. Hits 15 and 16 both show a significant reduction in fluorescence and therefore a reduction in the amount of RNA produced by RNA polymerase.

## 4 DISCUSSION

Phenotypic screening of large compound libraries is the current gold standard for the discovery of novel anti‐tubercular agents with promising pharmacokinetic properties.^32^, ^33^, ^34^ Large screens, such as these excel at finding novel inhibitors but fall short when it comes to target identification. This results in a reliance on WGS to identify the drug targets from spontaneous DRMs, which can be time consuming to generate. This process can often be complicated by promiscuous scaffolds that have little in the way of target specificity,^35^ or targets that have human homologues. Incorporating some aspect of target identification into preliminary screening can help alleviate these issues and accelerate the drug discovery process. In this work, we have developed a mCherry reporter screen to identify protein synthesis hits, more specifically inhibitors of RNA polymerase, tRNA synthase, and the ribosome.

*M*.* tuberculosis* protein synthesis is a well‐known druggable pathway with some of the most potent anti‐tubercular drugs targeting related enzymes. The rifampin class of antibiotics, most notably rifampicin, target RNA polymerase to halt the DNA to mRNA transcription.^4^, ^5^, ^6^, ^7^, ^8^ Ribosomal inhibitors, such as streptomycin, prevent the translation of mRNA into protein.^9^, ^10^, ^11^, ^12^, ^13^, ^14^ Additionally, mupirocin is a tRNA synthase inhibitor used topically for *Staphylococcus aureus*. While *M*.* tuberculosis* is intrinsically resistant to mupirocin,^36^ there are documented inhibitors of mycobacterial tRNA synthase.^20^, ^21^, ^22^, ^23^ Inhibition of tRNA synthase stops the regeneration of tRNA and prevents the addition of amino acids to nascent protein. The result of these forms of inhibition is a prevention of protein synthesis, resulting in cellular death.

The validation of the mCherry reporter assay has shown that cells expressing mCherry fluorescent protein subjected to known protein synthesis inhibitors display a dose‐dependent reduction of fluorescence as shown in Figure 3. The resazurin viability assay confirmed visually that the resulting reduction in fluorescence was not due to cellular death. This indicated that the fluorescence reduction stemmed only from a reduction in mCherry protein production.

The relationship between protein synthesis inhibitors and mCherry fluorescence has been optimized for a small‐scale proprietary screen and focused on a set of ribosomal inhibitors, previously untested against whole‐cell *M*.* tuberculosis*, in a dose‐response fashion. Two thirds of the hits showed a dose response consistent with patterns displayed by the known protein synthesis inhibitors during validation. In addition, quantitative measurement of resazurin fluorescence showed that the reduction was not due to cell death, suggesting that many of these inhibitors are effective at inhibiting the *M*.* tuberculosis* ribosome, as previously suggested by cell‐free assays. The advantage of using phenotypic methods, in particular with mycobacteria is that any hits found must be able to pass through the mycobacterial cell wall, meaning unsuccessful compounds are filtered out at an early stage of the drug discovery pipeline.

Based upon the success of the proprietary screen, the full GSK “TB box” was screened. This library of compounds is known to be effective against *M*.* tuberculosis*. Initially the full library was screened with a single shot approach. Hits were selected based upon a reduction of mCherry fluorescence to 50% or lower as shown in Figure 5 with viability confirmed using the quantitative resazurin assay. The 91 hits found were then progressed to dose‐response screening. Of the 91 hits, 18 displayed fluorescence decreases consistent with the known inhibitors used during validation. These 18 hits inhibit *M*.* tuberculosis* protein synthesis *via* an unknown mechanism of action and could potentially inhibit RNA polymerase, the ribosome or other involved enzymes, such as tRNA synthases.

Using an RNA polymerase assay we have shown that this screening method can correctly identify hits of the protein synthesis pipeline from compound libraries. Hits 15 and 16 both inhibit RNA polymerase *in vitro*. While they lack in potency when compared to the control compound rifampicin, these scaffolds could be valuable with further structural modification and optimization.

Presented herein, the developed screening method utilizes inducible mCherry fluorescent protein expression to rapidly screen compound libraries for inhibitors of the protein synthesis pipeline. The validation and subsequent screening of two sets of compounds have shown that the method is sensitive for selecting protein synthesis inhibitors from compound libraries. These hit compounds were subsequently examined with an RNA polymerase assay, which identified two inhibitors of RNA polymerase. While this screen excels at finding such hits, the method offers no means of distinguishing the different mechanisms of action that result in protein synthesis inhibition and therefore must be coupled with target identification assays. To capitalize on the success of this screening method, efforts are ongoing in our laboratory to develop a more robust fluorescent assay to test inhibitors against the ribosome to be coupled with the broken beacon RNA polymerase assay. We expect that this screen, combined with the RNA polymerase and ribosomal assays, can then be used to rapidly search for and confirm the target complex of protein synthesis inhibitors. In this regard, the newly developed screen can focus drug development efforts on narrow range of known effective targets.

## CONFLICT OF INTEREST

The authors declare no competing financial interests.

## AUTHOR CONTRIBUTIONS

Conceived and designed the experiments: CB, MJ, JC, GSB. Provided reagents: PM, RGR, JE, JL, XL, JS. Performed the experiments: CB, MJ. Analyzed the data: CB, MJ, GSB. Wrote the paper: CB, MJ, GSB.
